# Association of proteinuria and incident atrial fibrillation in patients with diabetes mellitus: a population-based senior cohort study

**DOI:** 10.1038/s41598-021-96483-5

**Published:** 2021-08-23

**Authors:** Juntae Kim, Pil-Sung Yang, Byoung-Eun Park, Tae Soo Kang, Seong-Hoon Lim, Sungsoo Cho, Su-Yeon Lee, Myung-Yong Lee, Gregory Y. H. Lip, Dongmin Kim, Boyoung Joung

**Affiliations:** 1 Division of Cardiology, Department of Internal Medicine, College of Medicine, Dankook University, 119, Dandae-ro, Dongnam-gu, Cheonan-si, Chungnam, 31116 Republic of Korea; 2 Department of Cardiology, CHA Bundang Medical Center, CHA University, 59, Yatap-ro, Bundang-gu, Seongnam, Gyeonggi-do 13496 Republic of Korea; 3 Division of Cardiology, Department of Internal Medicine, Yonsei University College of Medicine, 50 Yonseiro, Seodaemun-gu, Seoul, 03722 Republic of Korea; 4 Liverpool Centre for Cardiovascular Science, University of Liverpool and Liverpool Heart & Chest Hospital, Liverpool, UK

**Keywords:** Atrial fibrillation, Diabetes complications

## Abstract

Diabetes mellitus (DM) is considered an independent risk factor for atrial fibrillation (AF). The excess risk in relation to the presence of proteinuria has not been well elucidated. Our aim was to determine the association between the incidence of AF and proteinuria in diabetic population. A total of 240,499 individuals aged ≥ 60 years from the Korea National Health Insurance Service-Senior cohort from 2004 to 2014 were included. 4.2% of individuals with DM and 3.7% of controls were diagnosed with AF during a median follow-up period of 7.2 years. Amongst controls (participants without proteinuria and DM), DM only, proteinuria only, and DM with proteinuria groups, the crude incidences of AF were 0.58, 0.70, 0.96, 1.24 per 100 person-years respectively. Compared with controls, the weighted risk of AF was increased by 11% (hazard ratio = 1.11, 95% confidence interval = 1.02–1.20, P = .001), 48% (hazard ratio = 1.48, 95% confidence interval = 1.30–1.69, P < .001), and 66% (hazard ratio = 1.66, 95% confidence interval = 1.26–2.18, P < .001) in the DM only, proteinuria only, and DM with proteinuria groups, respectively (P for trend < .001). Degree of proteinuria in diabetic patients was associated with a significantly higher rate of incident AF in dose dependent manner. Thus, assessing proteinuria by a simple urine dipstick test could provide a useful adjunct to risk assessment for AF in elderly population with DM.

## Introduction

Atrial fibrillation (AF) is the most common sustained cardiac arrhythmia and is associated with a substantial risk of mortality and morbidity resulting from stroke, congestive heart failure, dementia, and impaired quality of life^1–3^. With an aging population, AF is likely to become an increasing public health burden^1^.

In addition to shared risk factors (e.g., hypertension and obesity), diabetes mellitus (DM) is considered an independent risk factor for incident AF^4–6^. The prevalence of AF is at least two-fold higher in patients with DM than in people without DM^7^. Patients with poorly controlled DM have a higher risk of AF and the development of adverse cardiovascular outcomes^8,9^. However, some studies have also reported that DM is not an independent risk factor for AF^10,11^. These discrepancies might result from heterogeneous groups included in DM patients, especially since the incidence of AF rises with increasing severity of microvascular complications (for example. retinopathy and renal disease)^12^.

Proteinuria is the earliest marker of kidney damage due to the progression of DM. In a community-based cohort study, proteinuria was independently associated with all-cause mortality and cardiovascular mortality^13^. However, the excess risk of AF in relation to proteinuria in patients with DM is less clear.

In this study, our aim was to determine the association between the incidence of AF and proteinuria detected using a urine dipstick test and second, the implications for clinical outcomes in participants with DM from an elderly population cohort. We investigated these associations in an elderly population with and without DM using the nationwide population-based National Health Insurance Service (NHIS)-Senior cohort (NHIS-Senior) database.

## Methods

### Data source

This study was based on the NHIS-Senior, which contains data from 558,147 individuals, ~ 10% of the entire elderly population in South Korea aged $\ge$ 60 years (~ 5.1 million) in 2002^14^. The NHIS-Senior database included the following information: sociodemographic and socioeconomic information, insurance status, health checkup examinations, and records of patients’ medical history. The complete cohort population was linked to Korean social security numbers, and all social security numbers were removed after constructing the cohort by providing serial numbers to protect individuals' privacy. This study was approved by the Institutional Review Board of Yonsei University Health System (4-2020-0839). The requirement for informed consent was waived by the Institutional Review Board of Yonsei University Health System because all analysis used anonymous data. The NHIS-Senior database used in this study (NHIS-2016-2-171) was created by the NHIS of Korea.

### Study population

**Figure 1:**
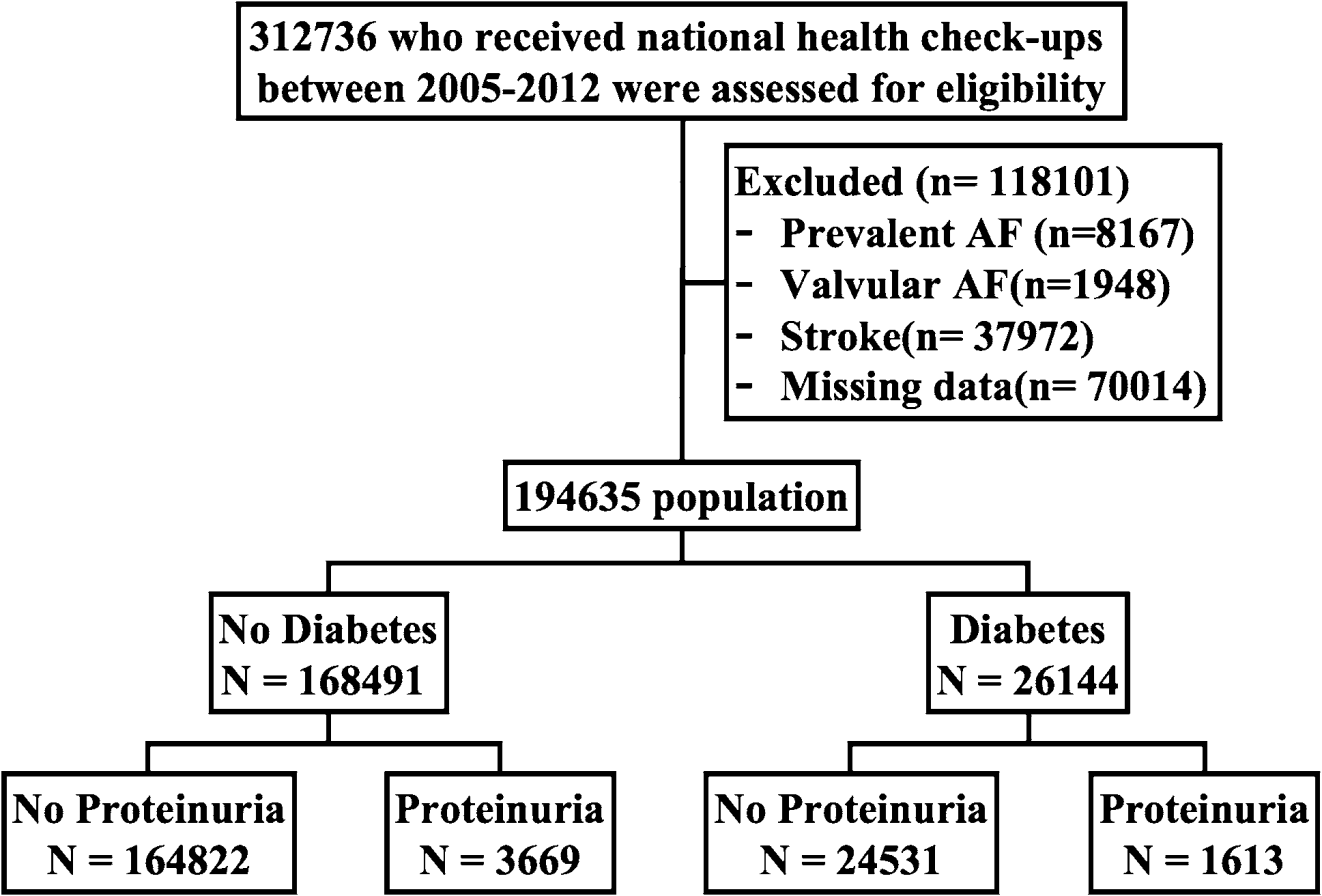
Flowchart of the study population enrollment and analyses.

From the Korean NHIS-Senior, a total of 312,736 patients who underwent a health checkup between 2005 and 2012 were enrolled, and follow-up data until December 2013 were reviewed. The exclusion criteria were as follows: (i) patients with AF before enrollment (n = 8873); (ii) AF with valvular heart disease, such as mitral valve stenosis and prosthetic valve disease (n = 1242); (iii) prior ischemic stroke or transient ischemic attack before enrollment (n = 39,069); (iv) prior hemorrhagic stroke before enrollment (n = 1342); (v) prior heart failure before enrollment (n = 18,786); and (vi) those who had missing values in the urine dipstick test at the 1st health checkup (n = 2925). Finally, we included 240,499 subjects, including 9107 with incident AF during the follow-up period (Fig. 1).

DM was defined using the International Classification of Diseases, Tenth Revision (ICD-10) diagnosis code E10-14 with concomitant prescription of all kinds of anti-diabetic drugs. The proteinuria threshold was defined as ≥ 1 + on the initial urine dipstick test. The predicted albumin-creatinine ratio (pACR) was calculated by the equation to convert urine dipstick protein to ACR^15^. The pACR was divided into 3 groups as A1 (< 30 mg/g), A2 (30 to 300 mg/g), or A3(> 300 mg/g), respectively.

### Covariates, and clinical outcomes

The sociodemographic variables included age, sex, and economic status. The baseline economic status was determined on the basis of the relative economic levels categorized into 11 levels ranging from group 0 (lowest) to group 10 (highest) according to their health insurance premiums paid, which directly reflect income levels of each individual. We obtained information on selected comorbid conditions from inpatient and outpatient hospital diagnoses. Baseline comorbidities were defined using the medical claims and prescription drug information prior to the index date. To ensure diagnostic accuracy, the patients were considered to have comorbidities when their condition was a discharge diagnosis or had been confirmed at least twice in an outpatient setting, in line with previous studies that used data from the NHIS database^2,3,14,16,17^. To evaluate the relationship between cardiovascular outcomes and proteinuria in diabetic population, we determined incident AF, stroke, heart failure hospitalization, and cardiovascular death using the ICD-10 code (eTable 1). The date of death, and ICD-10 code recorded for the death cause, was confirmed from the National Population Registry of the Korea National Statistical Office with unique personal identification numbers. The definition of AF diagnosis has been previously validated in the NHIS database with a positive predictive value of 94.1%^16,18^. We excluded AF patients with a diagnosis of valvular heart disease to limit the population to non-valvular AF patients. To evaluate de novo incidence of AF, stroke, heart failure hospitalization, and cardiovascular death, patients with prior recorded history of these cardiovascular outcomes before the index date were excluded. The follow-up period of each clinical outcome was defined as the time from the index date that participants enrolled in this study to the first occurrence of AF, stroke, heart failure hospitalization, or death or, end of the study period, whichever came first.

### Inverse probability of treatment weighting

To adjust potential systematic differences between different exposure groups, inverse probability of treatment weighting (IPTW) based on multinomial propensity scores was used to construct a weighted cohort of patients who differed with respect to DM or proteinuria but were balanced with respect to other measured characteristics^19^. In time-to-event analyses, IPTW minimizes bias relative to the other methods of applying propensity scores^20^. Also, the IPTW approach is suitable when comparing multiple groups^21^. The propensity scores were estimated by using generalized boosted models based on 10**,**000 regression trees. We adjusted for age, sex, and clinical variables, including hypertension, previous myocardial infarction, dyslipidemia, osteoporosis, chronic kidney disease, chronic obstructive pulmonary disease, liver disease, anemia, body mass index, alcohol, and smoking habits.

### Statistical analysis

Continuous variables are expressed as mean ± standard deviation (SD). Categorical variables are expressed as frequencies and percentages. The risk of incident AF associated with DM and proteinuria was compared using Kaplan–Meier analysis. The proportional hazard assumption was tested based on the Schoenfeld residuals. In the sensitivity analyses, we assessed the association between proteinuria, which was entered into the models as a time-varying factor, and each outcome using Cox proportional hazards regression models. The underlying time scale in these models was the observational period. The hazard ratios (HR) of AF for each combination of proteinuria change were calculated in Cox models using the consistently negative proteinuria group as the reference category. Urine dipstick results between the first and third tests were used to define changes in proteinuria. In these analyses, follow-up for AF was initiated from the third health examination (eFigure 1).

All tests were two-tailed, and P-values < 0.05 were considered statistically significant. Balance between different exposure groups was evaluated by standardized differences of all covariates, using a threshold of 0.1 to indicate imbalance. Statistical analyses were conducted using R Statistical Software/Environment (version 3.5.1, The R Project for Statistical Computing, Vienna, Austria).

### Ethics approval and consent to participate

This study was conducted in accordance with the ethical principles of the Declaration of Helsinki and was approved by the Institutional Review Board of Yonsei University College of Medicine, Seoul, Korea.

## Results

### Baseline characteristics

The baseline characteristics of the study cohort according to the categories of DM and proteinuria are summarized in Table 1. The mean age of study population was 71.0 ± 5.4 years (45% male). Patients with DM and/or proteinuria were older, had a higher body mass index, higher prevalence of hypertension, dyslipidemia, chronic kidney disease, and coronary artery disease than participants without DM and proteinuria. The median follow-up was 86 months (interquartile range [IQR], 58–95 months). After IPTW, all baseline characteristics had standardized differences less than 0.1 except fasting blood glucose, and estimated glomerular filtration rate (eGFR). (Table 1).

**Table 1:** Baseline characteristics.

	DM-Prot- (n = 202,291)	DM + Prot- (n = 31,088)	DM-Prot + (n = 4953)	DM + Prot + (n = 2167)	Maximum pairwise standardized mean difference*	
					Before IPTW	After IPTW
Mean age (SD), y	70.60 (5.42)	70.76 (5.05)	71.56 (5.89)	71.11 (5.17)	0.098	0.006
Male, n (%)	91,061 (45.0)	13,965 (44.9)	2309 (46.6)	1207 (55.7)	0.114	0.044
BMI (mean (SD))	23.55 (3.16)	24.47 (3.15)	23.95 (3.40)	24.59 (3.31)	0.187	0.018
**Smoking (%)**					0.092	0.044
No	151,150 (78.7)	23,566 (79.5)	3642 (77.1)	1519 (73.5)		
Former	14,636 (7.6)	2556 (8.6)	434 (9.2)	234 (11.3)		
Current	26,247 (13.7)	3531 (11.9)	647 (13.7)	315 (15.2)		
**Alcohol (%)**					0.06	0.044
Low (≤ 3/month)	146,146 (73.8)	23,546 (77.2)	3609 (74.3)	1587 (74.4)		
Moderate (1–2/week)	30,127 (15.2)	4204 (13.8)	709 (14.6)	342 (16.0)		
Heavy (≥ 3/week)	21,694 (11.0)	2748 (9.0)	542 (11.2)	204 (9.6)		
**Economic status (%)**					0.046	0.043
Low (0–4)	63,102 (31.2)	9246 (29.7)	1515 (30.6)	607 (28.0)		
Middle (5–8)	70,691 (34.9)	10,580 (34.0)	1715 (34.6)	761 (35.1)		
High (9–10)	68,498 (33.9)	11,262 (36.2)	1723 (34.8)	799 (36.9)		
Hypertension (%)	94,814 (46.9)	23,548 (75.7)	3051 (61.6)	1868 (86.2)	0.500	0.041
Dyslipidemia (%)	53,264 (26.3)	18,016 (58.0)	1592 (32.1)	1347 (62.2)	0.472	0.055
CKD or ESRD (%)	1733 (0.9)	668 (2.1)	152 (3.1)	140 (6.5)	0.167	0.007
History of MI (%)	2650 (1.3)	930 (3.0)	108 (2.2)	95 (4.4)	0.103	0.021
COPD (%)	13,528 (6.7)	2491 (8.0)	377 (7.6)	191 (8.8)	0.042	0.022
Liver disease (%)	41,201 (20.4)	9880 (31.8)	1079 (21.8)	684 (31.6)	0.168	0.047
Malignancy (%)	20,796 (10.3)	4149 (13.3)	593 (12.0)	297 (13.7)	0.060	0.024
CHD (%)	2783 (1.4)	1088 (3.5)	86 (1.7)	98 (4.5)	0.113	0.029
Sleep apnea (%)	101 (0.0)	27 (0.1)	5 (0.1)	0.0 (0.0)	0.026	0.027
Anemia (%)	33,586 (16.6)	6066 (19.5)	1066 (21.5)	666 (30.7)	0.177	0.033
Bleeding (%)	5216 (2.6)	1187 (3.8)	193 (3.9)	108 (5.0)	0.064	0.036
Coagulation or platelet defect (%)	2070 (1.0)	528 (1.7)	65 (1.3)	38 (1.8)	0.037	0.013
Venous thromboembolism (%)	1142 (0.6)	348 (1.1)	33 (0.7)	27 (1.2)	0.044	0.014
Hypothyroidism (%)	5137 (2.5)	1222 (3.9)	130 (2.6)	77 (3.6)	0.048	0.010
Hyperthyroidism (%)	4629 (2.3)	1173 (3.8)	129 (2.6)	98 (4.5)	0.073	0.010
Osteoporosis (%)	57,076 (28.2)	10,076 (32.4)	1290 (26.0)	556 (25.7)	0.083	0.022
Blood glucose (mg/dL)	97.2 ± 21.4	134.2 ± 51.3	103.7 ± 30.8	148.9 ± 59.1	0.717	0.763
Creatinine (mg/dL)†	0.95 ± 0.77	1.02 ± 1.09	1.14 ± 1.72	1.28 ± 1.02	0.168	0.066
eGFR (ml/min/1.73m^2^)†	73.7 ± 16.4	70.9 ± 17.9	67.1 ± 19.3	62.0 ± 22.3	0.336	0.162

Values are expressed in n (%), mean ± SD.

*BMI* body mass index, *CHD* coronary heart disease, *CKD* chronic kidney disease, *COPD* chronic obstructive pulmonary disease, *eGFR* estimated glomerular filtration rate, *ESRD* end-stage renal disease, *IPTW* inverse probability of treatment weighting, *MI* myocardial infarction, *SD* standard deviation.

*Proposed cut offs for acceptable standardized differences ranged from 0.1 to 0.25.

^†^Creatinine and eGFR were available in 19.7% of study population (N = 19,320).

### Risk for atrial fibrillation

Overall, 1410 (4.2%) individuals with DM and 7697 (3.7%) controls were diagnosed with AF during a median follow-up period of 7.1 years. In controls (participants without proteinuria and DM), DM only, proteinuria only, and DM with proteinuria groups, the crude incidences of AF were 0.58, 0.70, 0.96, and 1.24 per 100 person-years, respectively (Table 2). Subjects with DM and proteinuria had a higher cumulative incidence of AF than the unweighted (log-rank P < 0.001) and weighted group without DM and proteinuria (log-rank P < 0.001, eFigure 2 in the Supplement).

**Table 2:** Incidences of outcomes in the Study Cohort.

Group	Total patient, n	Event, n	Crude incidence per 100 patient-years	Weighted incidence per 100 patient-years	Absolute reduction in event rate (95% CI)	Weighted hazard ratio (95% CI)*	P for trend
**Atrial fibrillation**							< 0.001
DM-Prot-	202,291	7426	0.58	0.60		1.00 (reference)	
DM + Prot-	31,088	1269	0.70	0.65	0.12 (0.16–0.08)	1.11 (1.02–1.20)	
DM-Prot +	4953	271	0.96	0.88	0.38 (0.49–0.27)	1.48 (1.30–1.69)	
DM + Prot +	2167	141	1.24	1.00	0.66 (0.87–0.46)	1.66 (1.26–2.18)	
**Stroke**							< 0.001
DM-Prot-	202,291	9881	0.78	0.79		1.00 (reference)	
DM + Prot-	31,088	2277	1.27	1.29	0.69 (0.74–0.64)	1.67 (1.57–1.77)	
DM-Prot +	4953	303	1.08	0.99	0.49 (0.62–0.37)	1.31 (1.16–1.48)	
DM + Prot +	2167	254	2.30	2.36	1.72 (2.00–1.44)	3.02 (2.48–3.68)	
**Heart failure hospitalization**							< 0.001
DM-Prot-	202,291	3369	0.26	0.27		1.00 (reference)	
DM + Prot-	31,088	689	0.38	0.35	0.11 (0.14–0.08)	1.28 (1.15–1.43)	
DM-Prot +	4953	160	0.56	0.46	0.30 (0.38–0.21)	1.73 (1.45–2.06)	
DM + Prot +	2167	95	0.83	0.69	0.57 (0.73–0.40)	2.59 (1.93–3.46)	
**Cardiovascular death**							< 0.001
DM-Prot-	202,291	4458	0.34	0.35		1.00 (reference)	
DM + Prot-	31,088	815	0.44	0.46	0.10 (0.13–0.06)	1.40 (1.27–1.54)	
DM-Prot +	4953	193	0.67	0.54	0.32 (0.42–0.23)	1.57 (1.34–1.84)	
DM + Prot +	2167	102	0.87	0.77	0.53 (0.70–0.36)	2.44 (1.80–3.32)	

*After inverse probability of treatment weighting.

Covariates adjusted for age, sex, BMI, smoking, alcohol, hypertension, dyslipidemia, chronic kidney disease, myocardial infarction, chronic obstructive pulmonary disease, osteoporosis, liver disease, and malignancy.

As quantified by the clinical variable-adjusted HR, compared with controls, the risk of AF was increased by 5% (hazard ratio [HR] 1.05, 95% confidence interval [CI] 0.98–1.12, P = 0.144), 47% (HR 1.47, 95% CI 1.29–1.67, P < 0.001), and 75% (HR 1.75, 95% CI 1.47–2.08, P < 0.001) in the DM only, proteinuria only, and DM with proteinuria groups, respectively (P for trend < 0.001). Compared with controls, the IPT-weighted risk of AF was increased by 11% (HR 1.11, 95% CI 1.02–1.20, P < 0.001), 48% (HR 1.48, 95% CI 1.30–1.69, P < 0.001), and 66% (HR 1.66, 95% CI: 1.26–2.18, P < 0.001) in the DM only, proteinuria only, and DM with proteinuria groups, respectively (P for trend < 0.001) (Table 2).

**Figure 2:**
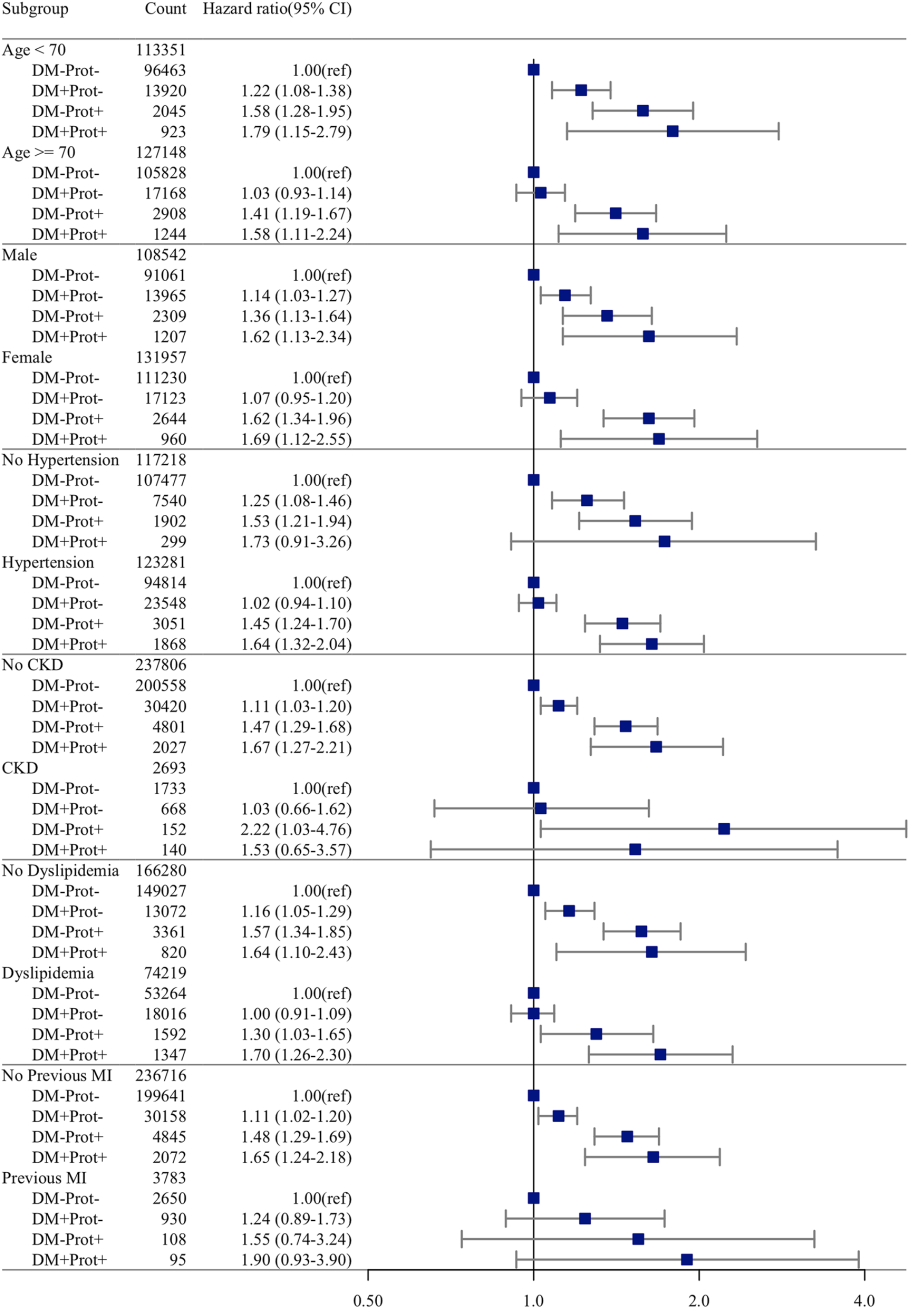
Hazard ratio for incident atrial fibrillation in different subgroups in overall population. Boxes indicate the hazard ratio, limit lines indicate the 95% confidence interval, and the vertical line (at hazard ratio 1) indicates no difference in the hazard ratios between atrial fibrillation and no atrial fibrillation.

In the subgroup analysis, the population with DM and proteinuria had a higher risk of AF incidence than the control group for all subgroups except subjects with chronic kidney disease, previous myocardial infarction or no hypertension. DM without proteinuria was associated with an increased risk of AF for subgroups without comorbidities. In patients with DM and hypertension, proteinuria was associated with an increased risk of AF (HR 1.75, 95% CI: 1.45–2.11, P < 0.001) (Fig. 2).

### Risk of stroke, heart failure hospitalization, and cardiovascular death

In the control, DM only, proteinuria only, and DM with proteinuria groups, the crude incidence of stroke was 0.78, 1.27, 1.08, and 2.30 per 100 person-years, respectively. Compared with controls, the IPT-weighted risk of stroke increased by 67% (HR 1.67, 95% CI 1.57–1.77, P < 0.001), 31% (HR 1.31, 95% CI 1.16–1.48, P < 0.001), and 202% (HR 3.02, 95% CI 2.48–3.68, P < 0.001) in the DM only, proteinuria only, and DM with proteinuria groups, respectively (P for trend < 0.001) (Table 2).

Compared with controls, the IPT-weighted risk of heart failure hospitalization was increased by 28% (HR 1.28, 95% CI 1.15–1.43, P < 0.001), 73% (HR 1.73, 95% CI 1.45–2.06, P < 0.001), and 159% (HR 2.59, 95% CI 1.93–3.46, P < 0.001) in the DM only, proteinuria only, and DM with proteinuria groups, respectively (P for trend < 0.001). Compared with controls, the IPT-weighted risk of cardiovascular death was increased by 40% (HR 1.40, 95% CI 1.27–1.54, P < 0.001), 57% (HR 1.57, 95% CI 1.34–1.84, P < 0.001), and 144% (HR 2.44, 95% CI 1.80–3.32, P < 0.001) in the DM only, proteinuria only, and DM with proteinuria groups, respectively (P for trend < 0.001) (Table 2).

### Risk of AF according to predicted albumin-creatinine ratio using dipstick test

All patients with a dipstick test of 1 + were classified using the pACR as having microalbuminuria. Only 367 patients who had trace results were classified with a pACR of 30–300 mg/g (eTable 2 in the Supplement).

**Figure 3:**
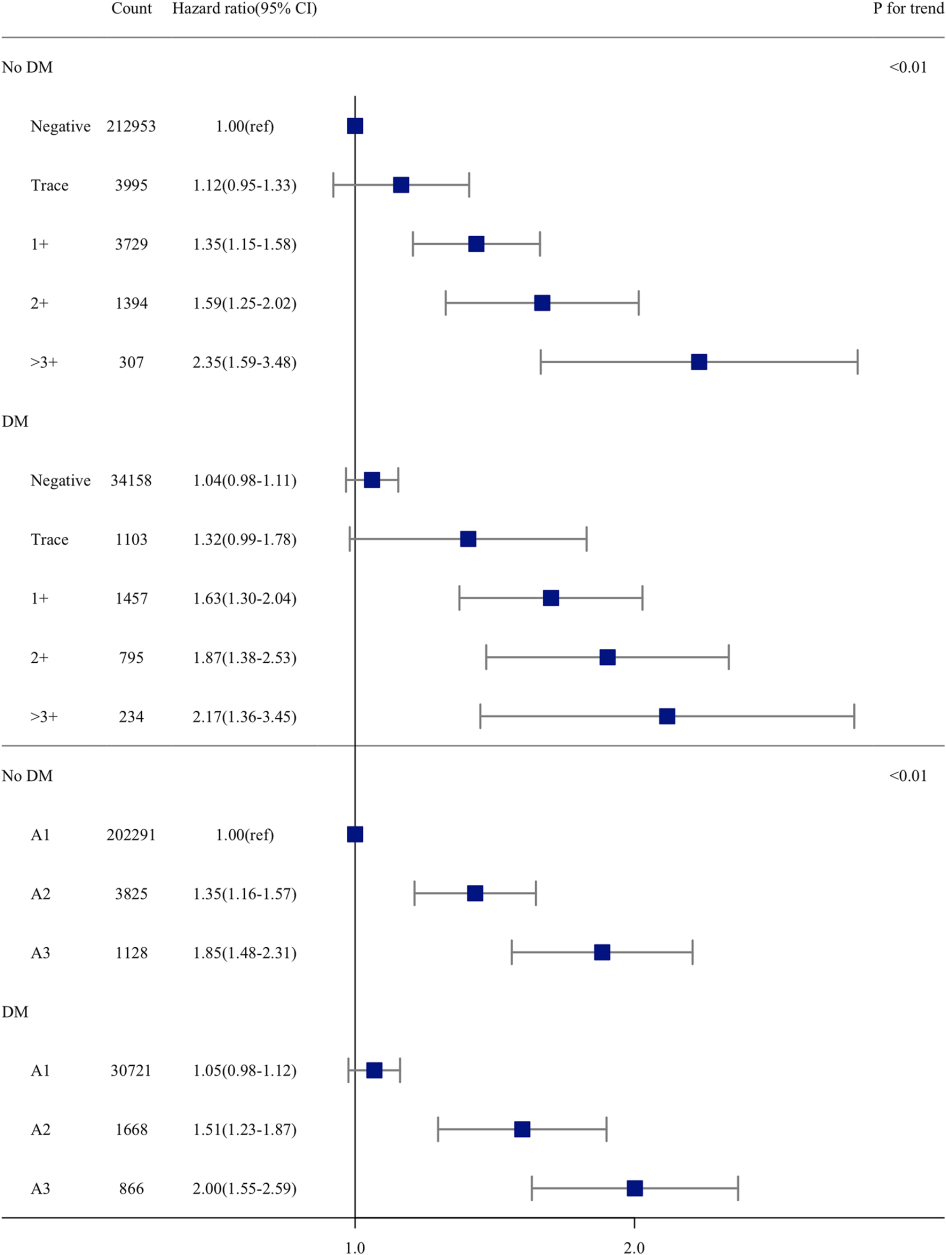
Hazard ratio for incident atrial fibrillation according to the degree of proteinuria by dipstick test and predicted albumin-creatinine ratio. Boxes indicate the hazard ratio, limit lines indicate the 95% confidence interval, and the vertical line (at hazard ratio 1) indicates no difference in the hazard ratios between atrial fibrillation and no atrial fibrillation. A1, predicted albumin-to-creatinine ratio, < 30 mg/g; A2, predicted albumin-to-creatinine ratio, 30–300 mg/g; A3 predicted albumin-to-creatinine ratio, > 300 mg/g.

Patients with a pACR representative of microalbuminuria and macroalbuminuria had higher HRs of AF than patients who had a pACR < 30 mg/g. Proportional increases in the risk of AF with the degree of proteinuria were found in diabetic and nondiabetic individuals (P for trend < 0.001) (Fig. 3).

### Risk of AF according to the change in proteinuria status

The change in proteinuria status was calculated among 45,800 individuals. There was a median interval of 3.8 (IQR, 3.4–4.1) years in patients who underwent a urine dipstick test between the 1st and 3rd health checkup (eFigure 1 in the Supplement). During the follow-up period, 908 incident AF events occurred. Subjects with persistent proteinuria had a higher cumulative incidence of AF than subjects who had negative results consistently (log-rank P < 0.001, eFigure 3 in the Supplement). Compared with those with consistently negative proteinuria, the risk of AF increased by 46% (HR 1.46, 95% CI 1.06–2.02, P = 0.021) and 175% (HR 2.75, 95% CI 1.67–4.53, P < 0.001) in the negative to positive and consistently positive groups, respectively. Patients who had resolved proteinuria had a similar risk of AF as that of the control group (HR 1.15, 95% CI 0.72–1.83 P = 0.569) (Table 3).

**Table 3:** Incidence of Atrial Fibrillation according to the follow-up dipstick test.

Change in proteinuria	Patients (n)	Events (n)	Incidence*	Absolute reduction in event rate (95% CI)	Hazard ratio (95% CI)
Negative → Negative	43,311	828	0.54		1 (ref)
Negative → Positive	1370	44	0.93	0.40 (0.37–0.43)	1.46 (1.06–2.02)
Positive → Negative	834	20	0.70	0.17 (0.13–0.20)	1.15 (0.72–1.83)
Positive → Positive	285	16	1.70	1.16 (1.08–1.25)	2.75 (1.67–4.53)

*per 100 Patient-Years.

Covariates adjusted for age, sex, BMI, smoking, alcohol, hypertension, dyslipidemia, chronic kidney disease, myocardial infarction, chronic obstructive pulmonary disease, osteoporosis, liver disease, and malignancy.

## Discussion

In this large, population-based, elderly cohort study, we demonstrated that proteinuria in diabetic individuals was associated with an increased risk of incident AF. Second, the risk of AF increased in a graded fashion with increasing proteinuria. Third, the resolution of proteinuria status was not significantly associated with an increased risk of incident AF. These findings have implications for measurement and therapeutic interventions to reduce proteinuria.

Previous studies have reported the association between DM and the risk of AF inconsistently^22–26^. However, the risk of AF was consistently elevated in advanced and complicated DM but not in simple DM. Dublin et al.^27^ reported that poorly controlled DM was associated with AF but well-controlled DM was not, and duration of DM was associated with AF. A recent meta-analysis suggested that higher serum HbA1c levels were associated with incident AF in prospective studies^28^. However, examination of this association was scarce in sub-populations with proteinuria in DM patients.

In this study using an elderly cohort, we demonstrated that the association between DM and AF, especially among participants with DM complicated with proteinuria. Although the risk of AF was increased in the overall patients with DM without proteinuria, the association was not consistent in different subgroups. In participants who were older or had comorbidities that increased risk of AF, DM without proteinuria did not significantly elevate the risk of AF. In contrast, elevated risk of AF was observed in DM with proteinuria group in most of the subgroups. Simple dipstick proteinuria screening can be utilized for the risk assessment of AF in patients with various subgroups.

In patients with DM, proteinuria is a hallmark of diabetic nephropathy. It remains the principal early predictor for decline in renal function^29^. Proteinuria in the diabetic population may represent poor glycemic control, longer duration of DM, and diabetic end organ damage. Also, patients with proteinuria had a higher prevalence of other risk factors for AF, such as hypertension, cardiovascular disease, and obesity. Proteinuria is a useful marker for predicting cardiovascular risk in patients with type 2 diabetic nephropathy^30^.

A urine dipstick test is less precise than quantitative laboratory measurements of albuminuria or proteinuria. However, these methods are preferred as initial screening tools for the evaluation of proteinuria because of their low cost, simplicity, and the ability to provide rapid point-of-care information^31^. Previous studies have shown that patients with proteinuria are associated with incident AF in the general population^33^. We also report a graded increase in the HR for AF with increasing dipstick proteinuria and ACR conversion. These findings were still significant independent of kidney function. Similar findings have been reported in previous studies^32^.

Interestingly, resolution of proteinuria status was not associated with the risk of incident AF compared with persistent or newly developed proteinuria. These findings highlight that time-dependent exposure to proteinuria can change the risk of AF and cardiovascular outcomes, and efforts to reduce proteinuria might be an invaluable method of preventing AF in patients with DM. Further studies are needed to determine whether interventions to help resolve proteinuria would decrease the risk of AF.

There is mounting evidence to support the influence of inflammation in the pathogenesis of AF^34^. Proteinuria may be a simple marker of endothelial dysfunction and chronic low-grade inflammation, which may lead to heightened atherogenetic states^35,36^. Glucose intolerance and insulin resistance may be associated with the development of the AF substrate, which is related to structural, electrical, electromechanical, and autonomic remodeling^37^. Diabetic nephropathy is associated with the renin–angiotensin–aldosterone system and lead to salt and water retention, causing left ventricular hypertrophy, and subsequent diastolic dysfunction with volume overload of the atria, which in turn may lead to AF by atrial dilatation^38^.

This study has several limitations. Although administrative databases are increasingly used for clinical research, such studies are potentially susceptible to errors arising from coding inaccuracies. To minimize this problem, we applied the definition that we had validated in previous studies using a Korean NHIS cohort^3,14,16^. Although the assessment of proteinuria using a dipstick has many advantages, it also has limitations, including only reflecting proteinuria in spot urine and low diagnostic accuracy. However, a report showed that dipstick proteinuria was more accurate in an elderly population with a higher prevalence of proteinuria than in the general population^39^. We could not exclude transient benign proteinuria and designate a specific cause of proteinuria. It should be noted that proteinuria in diabetic patients may be caused by factors unrelated to DM. Given the observational nature of this study, residual unidentified confounders may remain. To reduce this bias, we performed a sensitivity analysis using Cox proportional-hazards regression with IPTW and time-varying covariate (eTable 3 in the Supplement). Future studies are needed to determine whether preventing or delaying the progression of proteinuria using antihypertensive or diabetes medications can reduce the risk of incident AF. In addition, large prospective studies with data including albumin-creatinine ratio, HbA1c levels, and other biomarkers of cardiac and renal function are needed to clarify the effect of proteinuria in diabetic patients.

## Conclusions

DM was associated with an increased risk of AF, especially in patients with proteinuria. Proteinuria had adjunctive value for predicting AF. Thus, assessing proteinuria by a simple urine dipstick test could provide a useful adjunct to risk assessment for AF in elderly population with DM.

## Supplementary Information

Supplementary Information.

## Data Availability

The datasets used and/or analysed in the current study are available from the corresponding author on reasonable request.

## Abbreviations

AF: Atrial fibrillation
CI: Confidence interval
DM: Diabetes mellitus
HR: Hazard ratio
ICD: International Classification of Disease
IPTW: Inverse probability of treatment weighting
IQR: Interquartile range
pACR: Predicted albumin-creatinine ratio
SD: Standard deviation
