# Author Correction: Association of proteinuria and incident atrial fibrillation in patients with diabetes mellitus: a population-based senior cohort study

**DOI:** 10.1038/s41598-021-97510-1

**Published:** 2021-08-30

**Authors:** Juntae Kim, Pil-Sung Yang, Byoung-Eun Park, Tae Soo Kang, Seong-Hoon Lim, Sungsoo Cho, Su-Yeon Lee, Myung-Yong Lee, Gregory Y. H. Lip, Dongmin Kim, Boyoung Joung

**Affiliations:** 1 Division of Cardiology, Department of Internal Medicine, College of Medicine, Dankook University, 119, Dandae-ro, Dongnam-gu, Cheonan-si, Chungnam, 31116 Republic of Korea; 2 Department of Cardiology, CHA Bundang Medical Center, CHA University, 59, Yatap-ro, Bundang-gu, Seongnam, Gyeonggi-do 13496 Republic of Korea; 3 Division of Cardiology, Department of Internal Medicine, Yonsei University College of Medicine, 50 Yonseiro, Seodaemun-gu, Seoul, 03722 Republic of Korea; 4 Liverpool Centre for Cardiovascular Science, University of Liverpool and Liverpool Heart & Chest Hospital, Liverpool, UK

Correction to: *Scientific Reports* 10.1038/s41598-021-96483-5, published online 23 August 2021

The original version of this Article contained an error in the Funding section.

“This study was supported by a research grant from the Korean Healthcare Technology R&D project funded by the Ministry of Health and Welfare (HI15C1200, HC19C0130) and a CMB-Yuhan research grant of Yonsei University College of Medicine (6-2019-0124).”

now reads:

“This study was supported by a research grant from the Korean Healthcare Technology R&D project funded by the Ministry of Health and Welfare (HI15C1200, HC19C0130, HI19C0622) and a CMB-Yuhan research grant of Yonsei University College of Medicine (6-2019-0124).”

The original Article has been corrected.

