# Supplementary material for: Association of proteinuria and incident atrial fibrillation in patients with diabetes mellitus: a population-based senior cohort study
Source: Sci Rep. 2021 Aug 23;11:17013. doi: 10.1038/s41598-021-96483-5 (PMC8382825; doi:10.1038/s41598-021-96483-5)

**ONLINE SUPPLEMENT**

**Association of proteinuria and incident atrial fibrillation in patients with diabetes mellitus: A population-based senior cohort study**

**eTable 1.** Definitions and ICD-10 codes used for defining the comorbidities and clinical outcomes.

**eTable 2.** Predicted albumin-creatinine ratio and Urine dipstick test in KNHIS senior cohort

**eTable 3.** Hazard ratio of Cox proportional hazard models with time-varying covariate.

**eFigure 1.** Summary of the statistical analysis design.

**eFigure 2.** The cumulative incidence of atrial fibrillation.

**eFigure 3.** The cumulative incidence of atrial fibrillation according to the change of the proteinuria.

**eTable 1.** Definitions and ICD-10 codes used for defining the comorbidities and clinical outcomes.

|  | **Definitions** | **ICD-10 codes or conditions** |
| --- | --- | --- |
| **Comorbidities** |  |  |
| Proteinuria | Defined from laboratory data | Urine dipstick proteinuria 1+ or higher |
| Diabetes mellitus | Defined from diagnosis* plus treatment | ICD-10: E10, E11, E12, E13, E14  Treatment: all kinds of oral antidiabetics and insulin. |
| Atrial fibrillation | Defined from diagnosis* | ICD-10: I48 |
| Potential absence of non-valvular atrial fibrillation | Defined from any diagnoses of mitral stenosis or heart valve surgery | I05.0, I05.2, I34.2, Z95.2-4,  claim for valve replacement or valvuloplasty |
| Heart failure | Defined from diagnosis* | ICD-10: I11.0, I50, I97.1 |
| Hypertension | Defined from diagnosis* | ICD-10: I10, I11, I12, I13, I15 and antihypertensive medication |
| Dyslipidemia | Defined from diagnosis* | ICD-10: E78 |
| Ischemic stroke | Defined from diagnosis* | ICD-10: I63, I64 |
| Transient ischemic attack | Defined from diagnosis* | ICD-10: G45 |
| Hemorrhagic stroke | Defined from diagnosis* | ICD-10: I60, I61, I62 |
| Myocardial infarction | Defined from diagnosis* | ICD-10: I21, I22, I25.2 |
| Coronary heart disease | Defined from diagnosis and intervention | Acute myocardial infarction (ICD-10: I20x, I21x) and coronary angiography (HA670, HA680, HA681) |
|  | Coronary revascularization | PTCA (Percutaneous Transluminal Coronary Angioplasty): M6551, M6552,  PCI (Percutaneous Coronary Intervention; stent insertion): M6561, M6563, M6562, M6564  Percutaneous Transluminal Coronary Atherectomy: M6571, M6572  Percutaneous Thrombus Removal, Thrombolytic Treatment: M6634  Coronary artery bypass graft: O1641, OA641, O1642, OA642, O1647, OA647 |
|  | Defined from diagnosis* | I25.2 (Old myocardial infarction), I25.5 (Ischemic cardiomyopathy), I25.6 (Silent myocardial ischemia), I25.8 (Myocardial infarction as chronic), I25.9 (Chronic ischemic heart disease) |
| Chronic kidney disease | Defined from eGFR or diagnosis*  (if laboratory value was not available, diagnosis code was used) | eGFR <60mL/min per 1.73 m^2^  ICD-10: N18, N19 |
| End-stage renal disease | Defined from national registry for severe illness. | Patients with end-stage renal disease undergoing chronic dialysis or received a kidney transplant. |
| Sleep apnea | Defined from diagnosis* | ICD-10: G47.3 |
| Anemia | Defined from laboratory data | Hemoglobin concentration < 13g/dL in men and <12g/dl in women |
| Bleeding | Defined from diagnosis* | I60-I62, K25-28 (subcodes 0-2 and 4-6 only), K92.0, K92.1, K92.2, K62.5, I85.0, I98.3, N02, R31, R04, J942, K661, D62 |
| Coagulation or platelet defect | Defined from diagnosis* | D65, D66, D67, D68 (except D68.3), D69 (except D69.2) |
| Venous thromboembolism | Defined from diagnosis* | I80.1, I80.2, I80.3, I80.8, I80.9, I81, I82, I26 |
| Hyperthyroidism | Defined from diagnosis* | ICD-10: E05 |
| Hypothyroidism | Defined from diagnosis* | ICD-10: E03 |
| Hypothyroidism | Defined from diagnosis* | ICD-10: E03 |
| Chronic Liver disease | Defined from diagnosis of chronic liver disease, cirrhosis, and hepatitis | ICD-10: B18, K70, K71, K72, K73, K74, K76.1 |
| Chronic obstructive pulmonary disease | Defined from diagnosis* plus treatment | ICD-10: J42, J43(except J43.0), J44  Treatment: SABA, SAMA, LABA, LAMA, ICS, ICS+LABA, or methylxanthine (>1 months). |
| Malignancy | Defined from diagnoses of cancer (non-benign) | ICD-10: C00-C97 |
| **Clinical outcomes** |  |  |
| Atrial fibrillation | Defined from admission diagnosis or diagnosis at least twice in the outpatient department | ICD-10: I48 |
| Ischemic stroke | Defined from any discharge diagnoses with concomitant imaging studies | ICD-10: I63, I64 |
| Heart failure hospitalization | Defined from admission diagnosis (including only main and first sub-diagnosis) | ICD-10: I11.0, I50, I97.1 |
| Cardiovascular death | Defined from related death | ICD-10: I00-I78 |

*To ensure accuracy, comorbidities were established based on one inpatient or two outpatient records of ICD-10 codes in the database.

eGFR, estimated glomerular filtration rate; ICD-10, International Classification of Diseases-10th Revision.

**eTable 2.** Predicted albumin-creatinine ratio and Urine dipstick test in KNHIS senior cohort

| pACR | Dipstick test | Patient, n |
| --- | --- | --- |
| <30mg/g | Negative | 228779 |
| <30mg/g | Trace | 4233 |
| 30-300mg/g | Trace | 367 |
| 30-300mg/g | 1+ | 4649 |
| 30-300mg/g | 2+ | 477 |
| >300mg/g | 2+ | 1430 |
| >300mg/g | 3+ | 474 |
| >300mg/g | 4+ | 90 |

pACR, predicted albumin-creatinine ratio

**eTable 3.** Hazard ratio of Cox proportional hazard models with time-varying covariate

| Group | Hazard ratio(95% CI) | P for trend |
| --- | --- | --- |
| Atrial fibrillation |  | <0.001 |
| DM-Prot- | 1 (ref) |  |
| DM+Prot- | 1.04 (0.98-1.11) |  |
| DM-Prot+ | 1.64 (1.46-1.83) |  |
| DM+Prot+ | 1.83 (1.57-2.13) |  |
| Stroke |  | <0.001 |
| DM-Prot- | 1 (ref) |  |
| DM+Prot- | 1.56 (1.49-1.64) |  |
| DM-Prot+ | 1.36 (1.23-1.51) |  |
| DM+Prot+ | 2.55 (2.26-2.88) |  |
| heart failure hospitalization |  | <0.001 |
| DM-Prot- | 1 (ref) |  |
| DM+Prot- | 1.22 (1.11-1.33) |  |
| DM-Prot+ | 2.07 (1.80-2.39) |  |
| DM+Prot+ | 3.08 (2.57-3.7) |  |
| Cardiovascular death |  | <0.001 |
| DM-Prot- | 1 (ref) |  |
| DM+Prot- | 1.35 (1.25-1.47) |  |
| DM-Prot+ | 1.63 (1.42-1.87) |  |
| DM+Prot+ | 2.51 (2.09-3.02) |  |

Covariates adjusted for age, sex, BMI, smoking, alcohol, hypertension, dyslipidemia, chronic kidney disease, myocardial infarction, chronic obstructive pulmonary disease, osteoporosis, liver disease, and malignancy.

**eFigure 1.** Summary of the statistical analysis design. Black circles represent the enrollment of patients in this study cohort. Red line represent analysis according to the changes in proteinuria categories between the first and the third visit


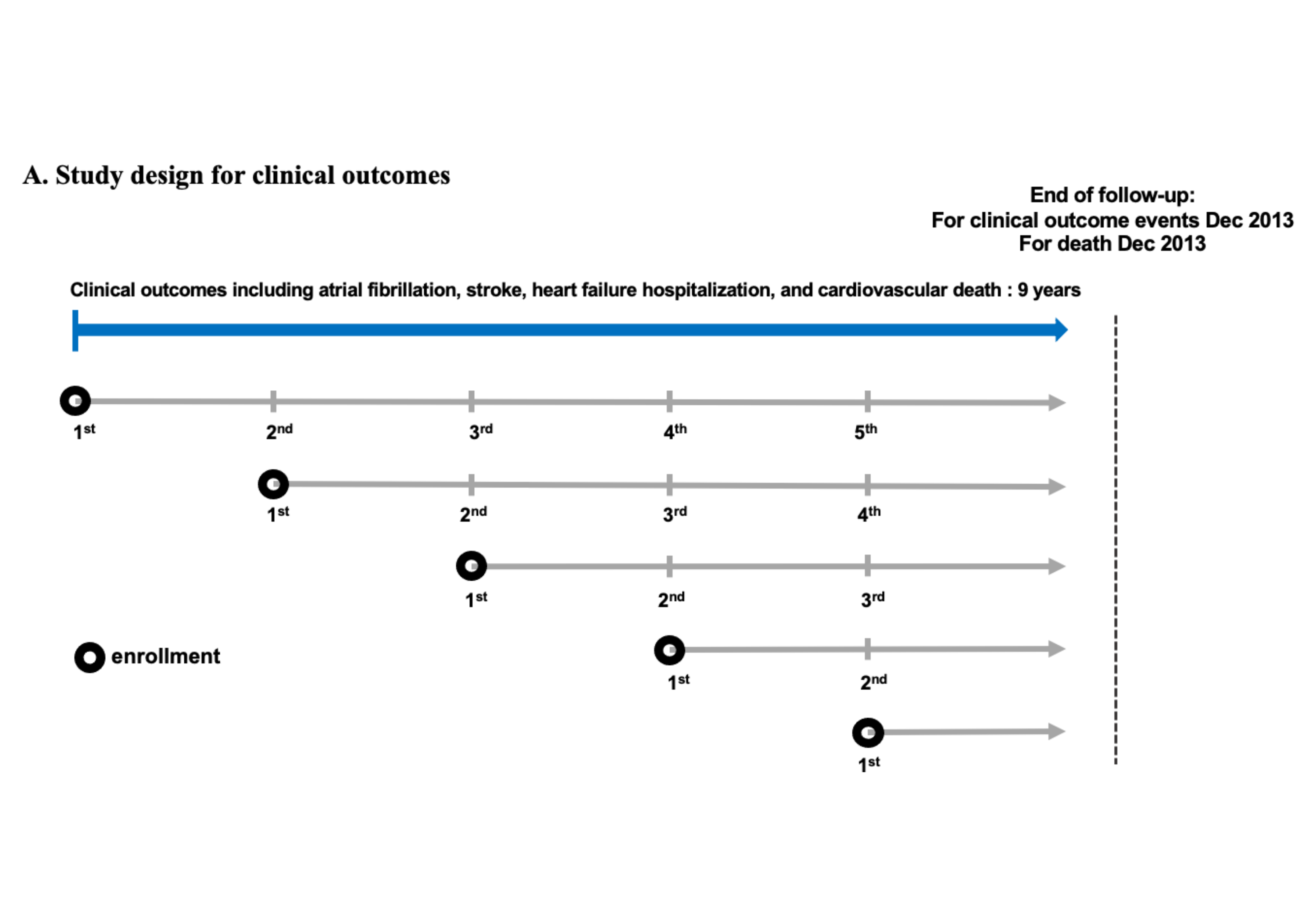

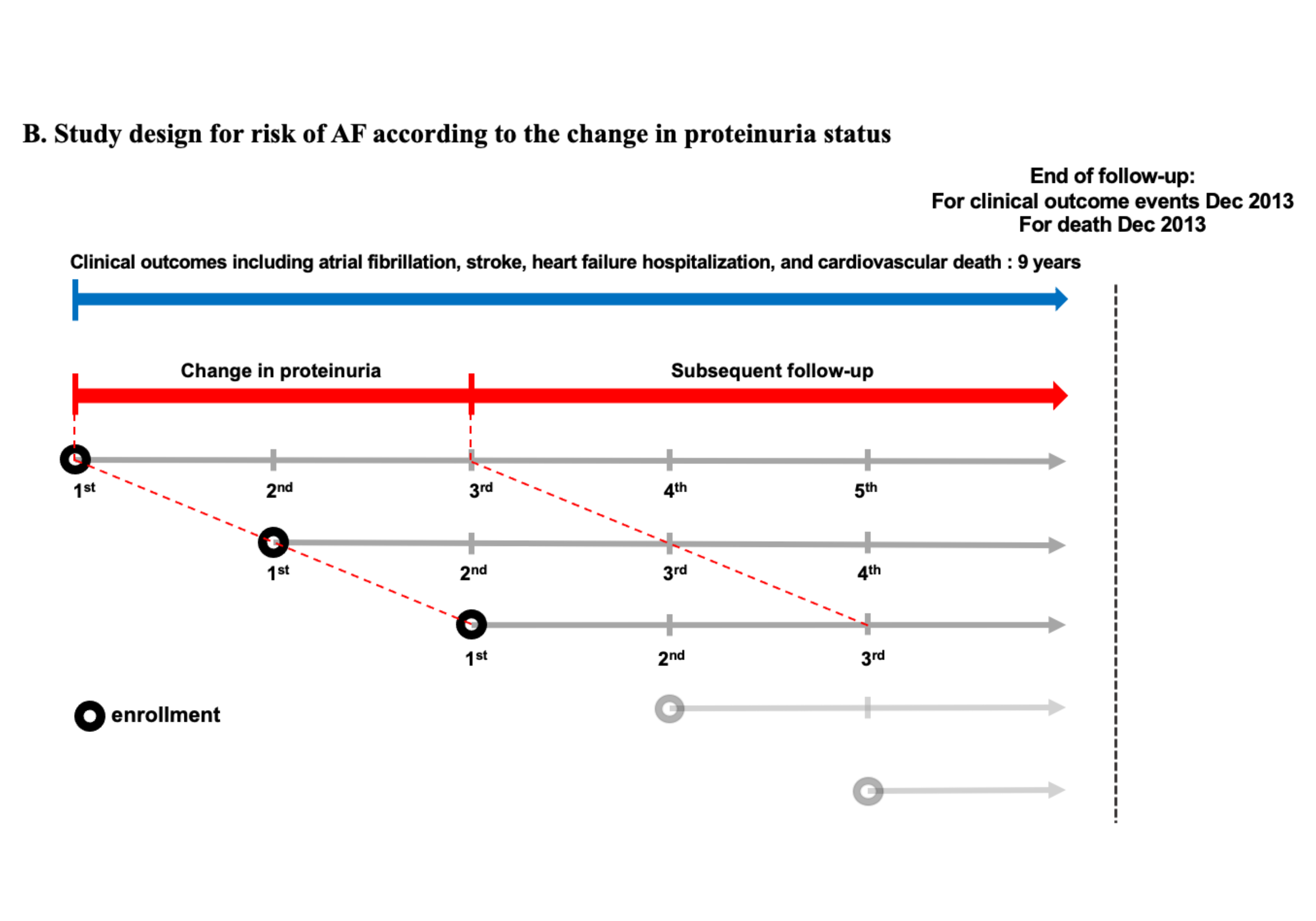


**eFigure 2.** The cumulative incidence of atrial fibrillation.


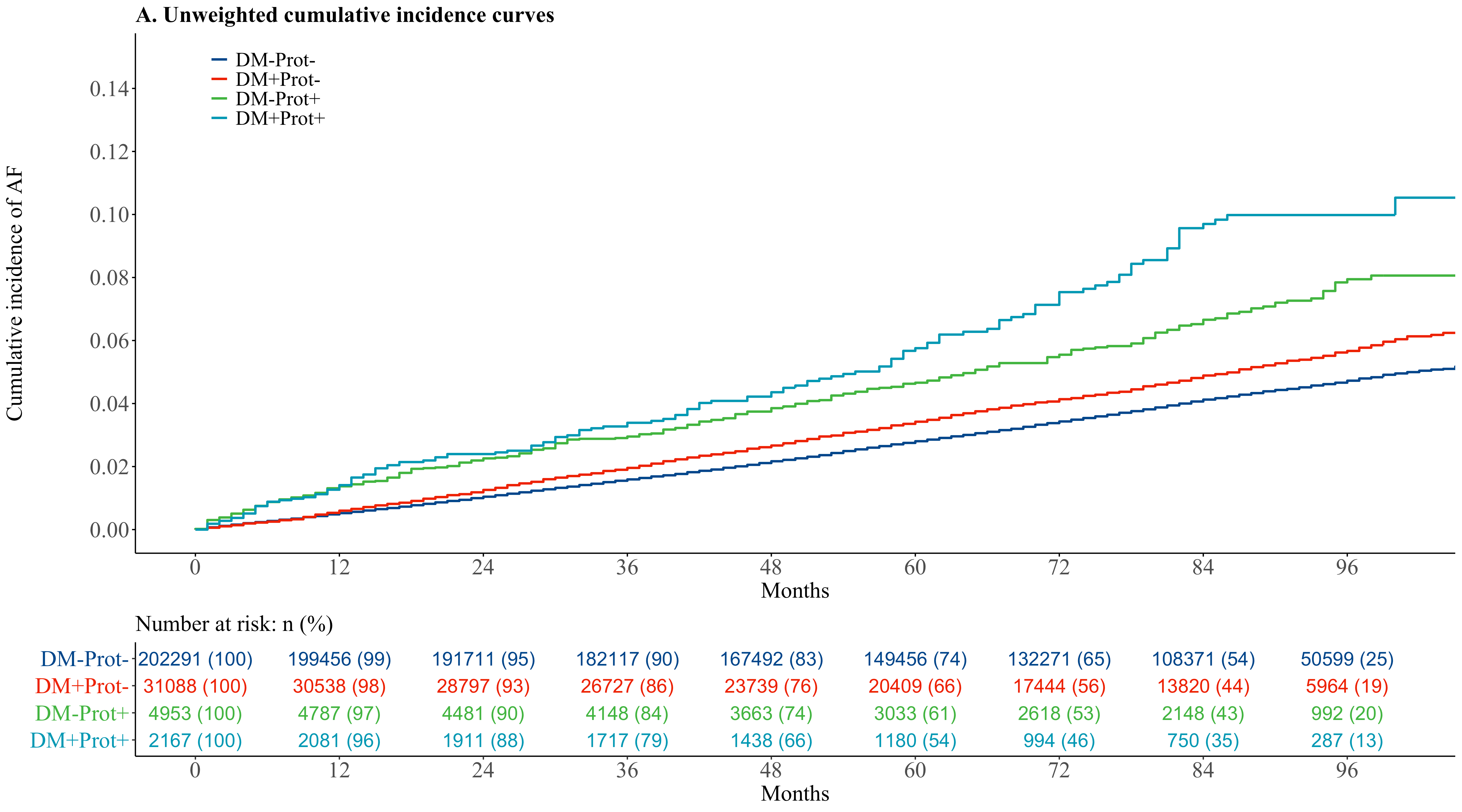


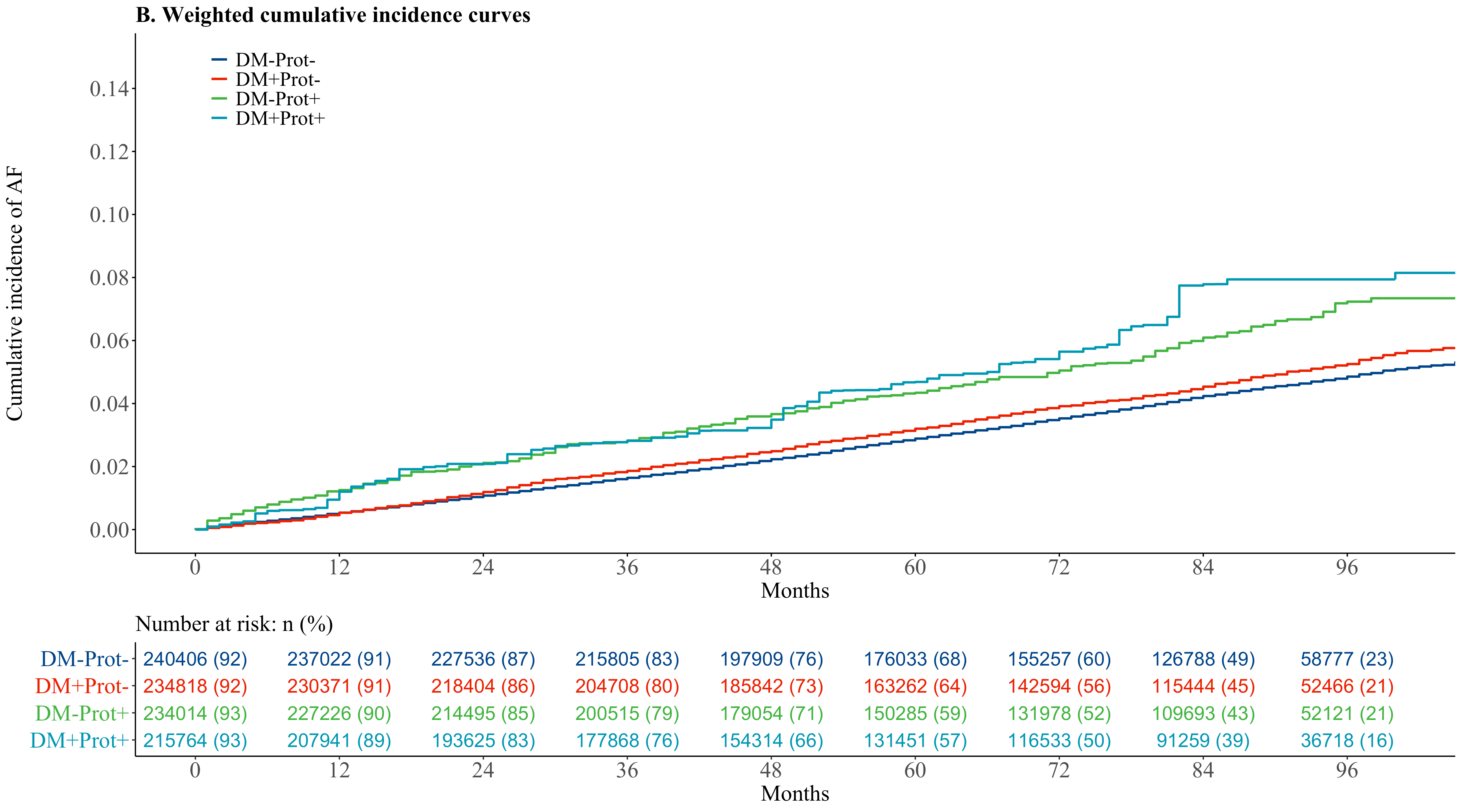


**eFigure 3.** The cumulative incidence of atrial fibrillation according to the change of the proteinuria.


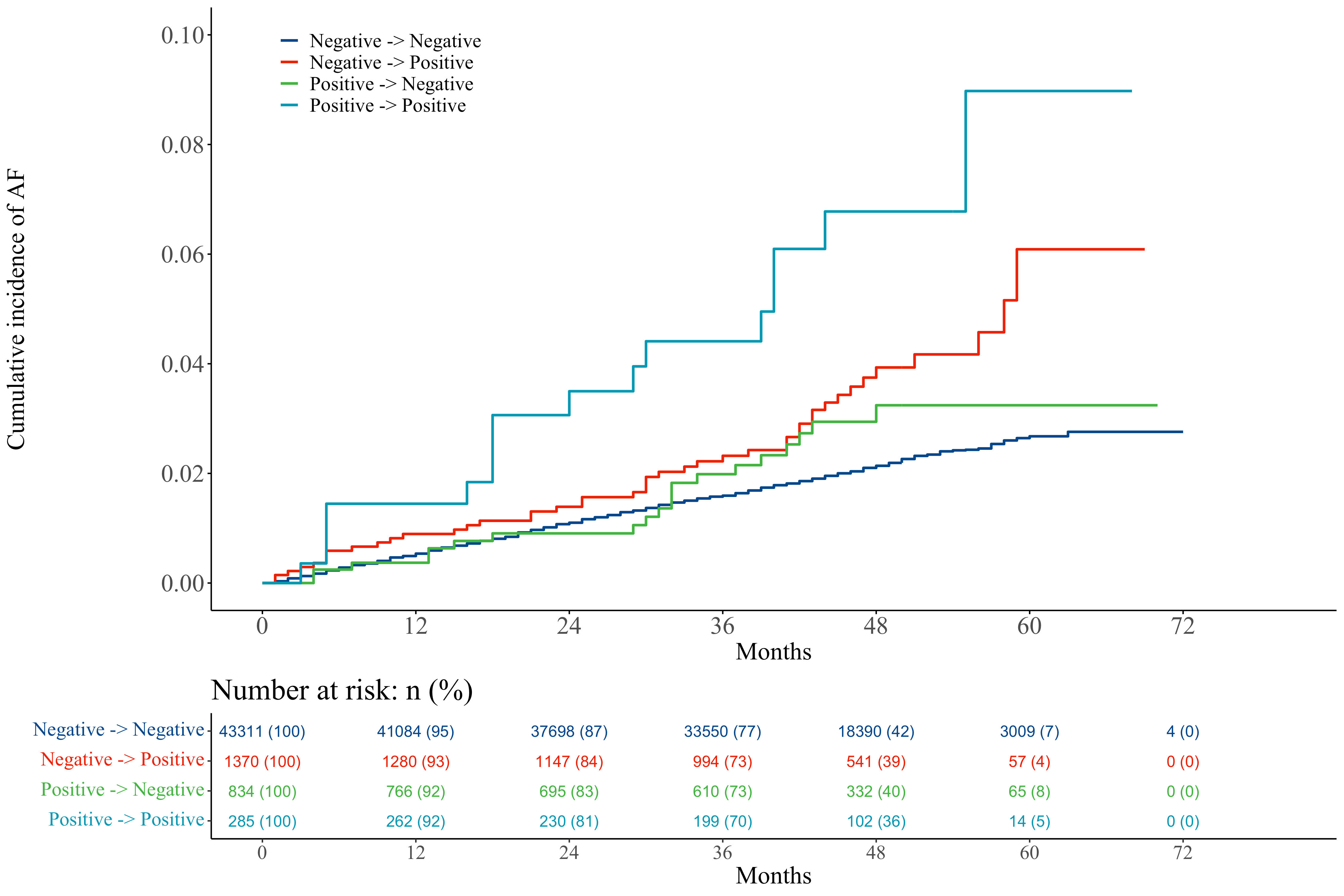

Supplement: Supplementary file 1 — Supplementary Information. [file 41598_2021_96483_MOESM1_ESM.docx]
